# Detection of the Mitochondrial Membrane Potential by the Cationic Dye JC-1 in L1210 Cells with Massive Overexpression of the Plasma Membrane ABCB1 Drug Transporter

**DOI:** 10.3390/ijms19071985

**Published:** 2018-07-07

**Authors:** Katarina Elefantova, Boris Lakatos, Jana Kubickova, Zdena Sulova, Albert Breier

**Affiliations:** 1 Institute of Biochemistry and Microbiology, Faculty of Chemical and Food Technology, Slovak University of Technology in Bratislava, Radlinského 9, 812 37 Bratislava 1, Slovakia; katarina.elefantova@stuba.sk (K.E.); jana.kubickova@stuba.sk (J.K.); 2 Institute of Molecular Physiology and Genetics, Centre of Bioscience, Slovak Academy of Sciences, Dúbravská cesta 9, 845 05 Bratislava 4, Slovakia; zdena.sulova@savba.sk

**Keywords:** multidrug resistance, P-glycoprotein, JC-1, mitochondrial membrane potential, tariquidar

## Abstract

JC-1, a cationic fluorescent dye when added to living cells, is known to be localized exclusively in mitochondria, particularly in good physiological conditions characterized by sufficient mitochondrial membrane potential (ΔΨ). The accumulation of JC-1 in these organelles leads to the formation J-aggregates (with a specific red fluorescence emission maximum at 590 nm), which is in addition to the typical green fluorescence of J-monomers (emission maximum of ∼529 nm). The lack of mitochondrial ΔΨ leads to the depression of JC-1 mitochondrial accumulation and a decrease in J-aggregate formation. Therefore, the ratio between the red and green fluorescence of cells loaded with JC-1 is often used for the detection of the mitochondrial membrane potential. However, JC-1 represents a suitable substrate of the multidrug transporter P-glycoprotein (P-gp). Therefore, the depression of the JC-1 content in intracellular space and particularly in the mitochondria to a level that is inefficient for J-aggregate formation could be expected in P-gp-positive cells. In the current paper, we proved this behavior on parental P-gp-negative L1210 (S) cells and their P-gp-positive variants obtained by either selection with vincristine (R) or transfection with the human gene encoding P-gp (T). P-glycoprotein inhibitors cyclosporine A and verapamil fail to restore JC-1 loading of the R and T cells to an extent similar to that observed in S cells. In contrast, the noncompetitive high affinity P-gp inhibitor tariquidar fully restored JC-1 accumulation and the presence of the typical red fluorescence of J-aggregates. In the presence of tariquidar, measurement of the JC-1 fluorescence revealed similar levels of mitochondrial membrane potential in P-gp-negative (S) and P-gp-positive cells (R and T).

## 1. Introduction

The assessment of mitochondrial membrane potential (ΔΨ) in intact cells could yield information necessary for the evaluation of their physio-pathological conditions [1]. The loss of mitochondrial membrane potential often takes place during the induction of neoplastic cell death induced by appropriate drugs [2]. When JC-1 (a cationic dye) is applied to intact cells, it is known to be predominately localized in mitochondria and represents a reliable fluorescent probe for the assessment of their mitochondrial ΔΨ [3]. This dye exhibits fluorescence emission at two typical wavelengths (both excited at 488 nm): (i) red fluorescent J-aggregates (emission maximum at 590 nm) at higher mitochondrial concentrations reflecting higher mitochondrial potential; and (ii) green fluorescent J-monomers (emission maximum at ~529 nm) at lower mitochondrial concentrations indicating lost membrane potential [4]. The ratio of the red/green fluorescence is independent of the mitochondrial shape, density, or size, and it depends only on the membrane potential. It has been used for detection of whether all of the mitochondria in the same cell have a uniform membrane potential using confocal microscopy [4]. JC-1 could also be used in fluorescent flow-cytometry studies of the mitochondrial membrane potential of living cells [5].

Neoplastic cells, including blood malignancies, could develop a multidrug resistance (MDR) phenotype, in which cells manifest dramatically improved cell resistance towards a large group of toxic substances with different structures, and the mechanism of cell death has effects on induction [6]. The major mechanism of multidrug resistance is associated with the efflux activities of plasma membrane transporters that belong to the family of ABC (ATP-binding cassette) transporters (reviewed in [7,8]). MRP1-3 (multidrug resistance-associated proteins, also known as ABCC1-3 proteins), BCRP (breast cancer resistance protein also known as ABCG2 protein) and particularly P-gp (P-glycoprotein also known as ABCB1 or MDR1 protein) have often been described to be a molecular cause of MDR development in neoplastic cells [9]. These transporters are known to be responsible for dramatically reduced cell sensitivity to a large group of structurally unrelated cytotoxic drugs [10]. Additionally, several intracellular fluorescent dyes could fulfill the criteria to be substrates for these transporters [11]. Therefore, these transporters may represent a real obstacle for trouble-free application of fluorescent indicators for their known analytical utilization in the characterization of MDR neoplastic cells by fluorescent flow cytometry and/or confocal microscopy [5]. In addition to other fluorescent intracellular indicators, JC-1 was also identified as substrate of P-gp that may be used for the sensitive measurement of P-gp efflux activity in intact cells [12,13]. The cellular efflux of JC-1 secured by P-gp may reduce its cellular content to an extent in which this indicator is not present in mitochondria in a sufficient amount and consequently cannot form red fluorescent J-aggregates in these organelles [14]. This may be untruly considered to be the loss of mitochondrial membrane potential in P-gp-positive cells. Therefore, P-gp efflux activity in P-gp-positive cells must be inhibited by an appropriate inhibitor for securing their sufficient loading with JC-1 that is necessary for accurate ΔΨ measurements. The current paper is dealing with the study of differences in the effects of known P-gp inhibitors (verapamil, VER; cyclosporine A, CSA; and tariquidar, TQR) on the JC-1 loading and fluorescence in P-gp-positive and P-gp-negative cells. The following variants of mice leukemia L1210 cells were used as cell models: (i) P-gp-negative parental cells (S); (ii) P-gp-positive-R cells obtained by the selection of S cells in medium containing vincristine (VCR) in stepwise increasing concentrations [15]; and (iii) P-gp-positive T cells obtained by the transfection of S cells with the gene encoding full length P-gp [16].

## 2. Results

### 2.1. Characterization of S, R and T Variants of L1210 Cells

Both P-gp-positive variants of L1210 cells (R and T) express a massive amount of either *ABCB1* mRNA encoding P-gp or P-gp protein (Figure S1A in the supplementary materials). In contrast, S cells did not express the *ABCB1* gene in mRNA and/or protein levels. Using immunofluorescence confocal microscopy, we found the predominant part of the P-gp protein localized on the cell surface in R and T cells, consistent with the generally accepted P-gp cellular localization in the plasma membrane (Figure S1B in the supplementary materials). A small amount of P-gp is also visible in the intracellular compartments of R and T cells. S cells did not contain any visible amount of P-gp either on the cell surface or intracellular space. All of these characteristics were periodically controlled during the time when the experiments for this paper took place.

### 2.2. Measurement of JC-1 Fluorescence in S, R and T Cells by Fluorescence Cytometry

**Figure 1:**
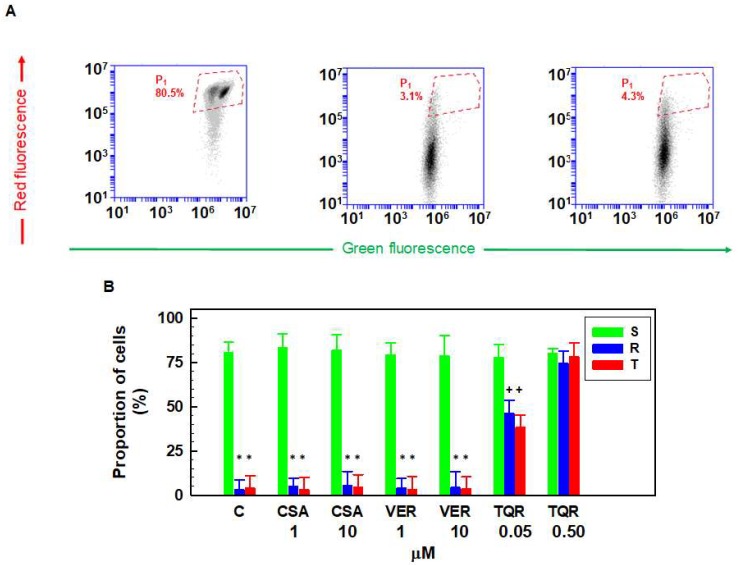
Detection of JC-fluorescence in S, R and T cells loaded with JC-1 in the absence or presence of P-gp inhibitors verapamil (VER), cyclosporine A (CSA) and tariquidar TQR. (**A**) Differences in the JC-1 signal between P-gp-negative (S) and P-gp-positive (R and T) cells measured by flow cytometry. The areas where the predominant proportions of S cells are present on the dot plots are bordered with red dashed lines. Data are representative for three independent measurements. Similar measurements as in (**A**) were also performed for cells influenced with VER, CSA (at concentrations of 1 and 10 μM) and TQR (in a concentration range of 0.05–0.50 μM). In the case of TQR, the control experiment was measured in the same concentration of dimethyl sulfoxide that was applied for TQR addition; (**B**) Effect of P-gp inhibitors on the JC-1 signal in S, R and T cells. The proportions of cells in the red-bordered areas in (**A**) are documented in the column plots. Data represent the mean ± SD from three independent measurements. Significance: * data differ significantly from the corresponding measurement obtained for S cells at the level *p* < 0.001; + data differ significantly from the corresponding measurement obtained for S cells at the level *p* < 0.01.

Double fluorescence staining of mitochondria by JC-1, either as green fluorescent J-monomers or as red fluorescent J-aggregates, was used for monitoring the mitochondrial membrane potential by flow cytometry (Figure 1A). More than 90% of all cells formed a homogenous cell population according to the evaluation using forward and side scatter, and this population was used for further cytometry documentation. The predominant proportion (80.5%) of S cells was intensively stained by the fluorescence of both J-aggregates and J-monomers, and in the respective dot plots, they were localized in area P_1_ bordered by the red dashed line in Figure 1A. According to Accuri cytometers application note [17], for JC-1 measurement of the mitochondrial membrane potential, cytometric data have to be compensated. Eighteen percent compensation was detected as optimal for S, R and T cells (as is documented for S cells in the Supplementary materials in Figure S2). 

In contrast, P-gp-positive R and T cells contained considerably less double stained cells and only 3.1% and 4.3% were localized in the area typical for S cells (Figure 1A). This may be caused by two reasons: (i) the mitochondrial membrane potential in P-gp-positive L1210 cells (R and T) achieved lower levels than in S cells, and therefore, R and T cells were less intensively stained by red fluorescent J-aggregates; and (ii) JC-1, as a P-gp substrate, is effectively eliminated from the internal space of P-gp-positive cells, and therefore, it could not be localized in the mitochondria in a sufficient amount for double staining. To resolve whether the first or second possibility was valid, we had to apply inhibitors of P-gp efflux activity. Two of the most frequently used inhibitors of P-glycoprotein, VER and CSA, failed to restore JC-1 double staining in R and T cells to a similar extent as that shown for S cells (Figure 1B). In contrast, another P-gp inhibitor TQR at a concentration of 0.5 μM was able to restore JC-1 double fluorescence staining in R and T cells (Figure 1B). A higher concentration of TQR did not induce additional effects on JC-1 double staining of R and T cells. The presence of VER, CSA, and TQR did not alter typical JC-1 double staining in S cells. 

**Figure 2:**
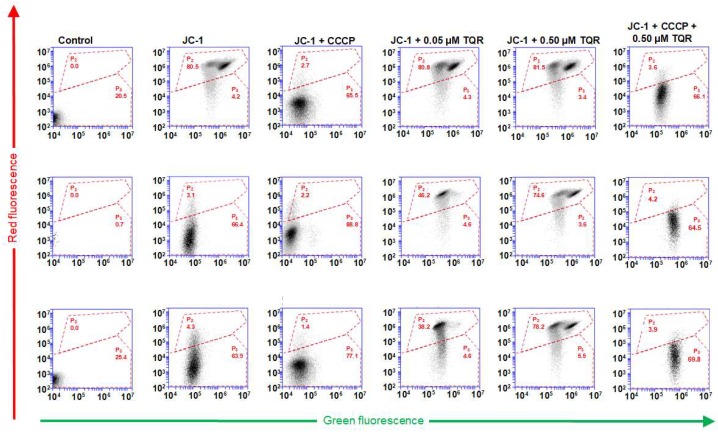
Measurement of JC-fluorescence in S, R and T cells loaded with JC-1 in the absence or presence of tariquidar and carbonyl cyanide 3-chlorophenylhydrazone. Data were evaluated using 18% compensation, which was detected as optimal (Figure S2, Supplementary materials). Typical double stained cells are present in area P_2_, and cells with limited staining are found in area P_3_, both bordered by red dashed lines. The data are representative of three independent measurement.

The proportions of JC-1 double stained S, R and T cells obtained in the absence or presence of 0.05 and 0.50 μM concentrations of TQR and in the absence or presence of CCCP (carbonyl cyanide 3-chlorophenylhydrazone; 50 μM) are summarized in Figure 2. An Accuri application note [17] recommended zooming in for the dot plots of FL1 in the region 10^4^–10^7^ and for FL2 in the region 10^2^–10^7^ to obtain precise gating of the double stained cells in area P_2_ against more than 80% of typically double stained S cells that were registered in this area. The diminution of the mitochondrial membrane potential by CCCP (at the recommended concentration of 50 μM) induced a strong reduction of double stained S cells to a value not exceeding 5% in P_2_. The population of S cells with reduced mitochondrial membrane potential was used for cell gating in area P_3_. In contrast to S cells, only negligible amounts (less than 5%) of R and T cells were double stained by JC-1 (visible in area P_2_), and the addition of CCCP did not greatly change this feature. However, TQR at a concentration of 0.50 μM ensured an almost identical proportion of double stained R and T cells (more than 70% in area P_2_) as was observed for S cells (Figure 2). No visible changes in the double staining of S cells were observed after treatment with TQR at 0.05 and 0.50 μM concentrations. Therefore, we could conclude that TQR at a concentration of 0.50 μM fully restored R and T cell double staining by JC-1. However, this P-gp inhibitor at a lower concentration (0.050 μM) induced only partial restoration of the double staining of P-gp-positive cells by JC-1 to values of 46% and 38% for R and T cells, respectively (Figure 2). The application of TQR (0.5 μM) together with CCCP (50 μM) induced similar depression of J-aggregate fluorescence in all three variants of L1210 cells [17]. All of the above facts indicated that P-gp protected R and T cells against loading with JC-1 and this could be reversed by TQR as a P-gp inhibitor.

### 2.3. TQR as a Reversal Agent of P-gp-Mediated Drug Resistance in R and T Cells 

To prove whether TQR can reverse P-gp-mediated MDR in R and T cells, we measured the effect of TQR on cell death effects induced by VCR, as a P-gp prototypical cytotoxic substrate, [18] on S, R and T cells (Table 1). Vincristine (0.25 μM) induced a massive cell death effect in S cells. In contrast, the viability of either R or T cells remained practically unchanged by this dose of VCR, indicating resistance to this substance. TQR can partially reverse the resistance of R and T cells to VCR at a concentration of 0.05 μM and completely at a concentration of 0.50 μM (Table 1). 

On the other hand, TQR alone at concentrations of 0.050 and 0.50 μM did not alter the cell viability of all three variants of L1210 cells significantly (Table 1). Thus, TQR did not considerably induce pronounced cell death effects on L1210 cells regardless of whether P-gp is present or absent. This may be additionally documented by measurements of apoptosis or necrosis progression using cell double staining with annexin V linked with fluorescein isothiocyanate is and propidium iodide (Figure S3, Supplementary materials) in a flow cytometer. Any marked changes in the proportion of either living cells or cells entering death progression by the apoptotic and necrotic mode were not visible in this measurement. 

**Figure 3:**
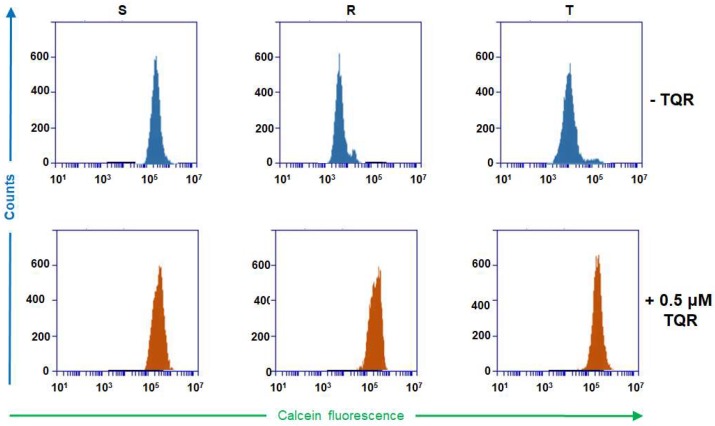
Recording of Calcein retention within S, R and T cells in the presence or absence of tariquidar (TQR). Calcein retention assays were performed in the absence and presence of TQR (at a concentration of 0.50 μM). The data are representative of three independent measurements.

We proved the TQR inhibitory effect on P-gp efflux activity by using the typical Calcein/AM retention assay [19]. Massive retention of Calcein is visible within S cells (Figure 3). In contrast, the retentions of Calcein within R and T cells are much less pronounced. TQR at a concentration 0.5 μM restored Calcein retention to a similar extent as is observed for S cells. These data indicated that a concentration of TQR of 0.5 μM is sufficient for the effective blockage of P-gp efflux activity, and a higher concentration of TQR did not induce any additional improvement of Calcein retention within R and T cells. Moreover, the TQR range did not induce any significant changes in Calcein retention within S cells.

### 2.4. Confocal Microscopy Detection JC-1 Labeling of S, R and T Cells 

**Figure 4:**
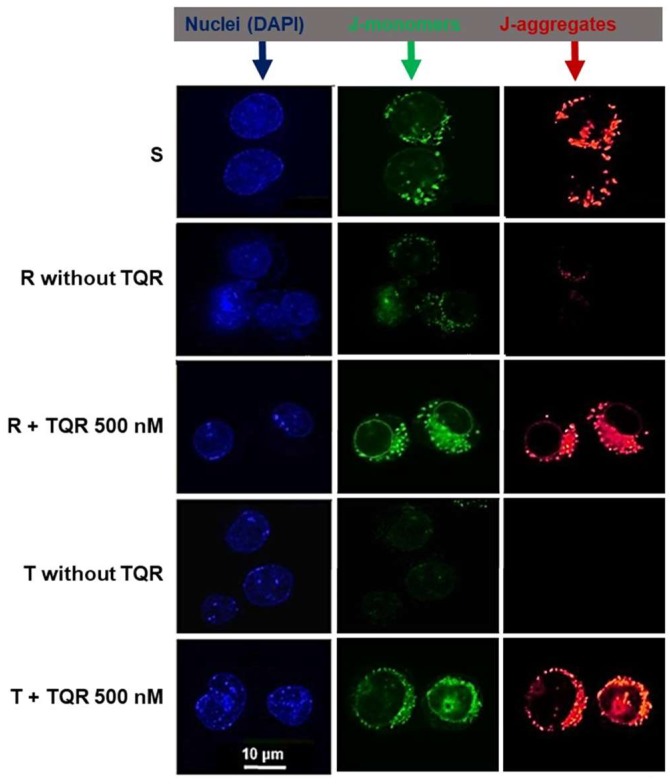
Detection of JC-1 signals in S, R and T cells by fluorescence confocal microscopy. The double staining of cells by JC-1 is visible either as green for J-monomers or red for J-aggregates. Data are representative for three independent measurements. Cells were labeled by DAPI to visualize nuclei and JC-1 to visualize mitochondria. Fluorescence was registered using excitation at 488 nm and adjusting the emission of confocal microscopy for 4′,6-diamidino-2-phenylindole (DAPI; used for nuclei staining and visible as blue), J-monomers (visible as green) and J-aggregates (visible as red/orange). TQR—tariquidar.

We used confocal microscopy detection of J-aggregates and J-monomers in native cells after their incubation with JC-1 alone (S, R, and T cells) or given together with TQR at a concentration 0.5 μM (R and T cells) to observe whether the populations of the mitochondria of cells are rather uniform or differ in membrane potential. S cells contain mitochondria labeled with the fluorescence typical for both J-aggregates and J-monomers (Figure 4). 

Both fluorescences are localized in small subcellular particles in cellular space distinct from the nuclei labeled by DAPI, which is consistent with mitochondrial labeling. J-aggregates and J-monomers were found in cells in the same locations, thus indicating the uniformity of the mitochondria from the point of the mitochondrial membrane potential. In contrast, the labeling of R and T cells by JC-1 alone is much less pronounced, particularly when the fluorescence for the J-aggregates is registered. However, when TQR was added together with JC-1, a similar staining of cells to that in S cells was obtained.

## 3. Discussion 

P-gp glycoprotein is known to transport a large group of structurally unrelated substances, known as P-gp substrates, out from inner cellular space [20]. In addition to different anticancer drugs, several intracellular fluorescent probes including the intracellular calcium indicators fura-2/AM [21,22], fluo-3/AM [22,23] and Calcein/AM [19] or mitochondria-labeling substances such as MitoTracker Green [24] and JC-1 [3,13,14] belong to the group of this ABC transporter substrates. P-gp operates against cell loading by these fluorescent probes in P-gp-positive cells, which reduces their intracellular retention. This was responsible for the difficulty in the measurement of eventual differences in intracellular calcium levels between R and T cells by calcium indicators, and therefore, this issue was measured radiometrically using ^45^Ca^2+^ [25,26]. Due to the P-gp efflux activity, strong alterations of mitochondrial staining by mitochondrial fluorescent markers such as MitoTracker Green or JC-1 have to be expected when P-gp-negative and P-gp-positive cells are to be compared. This may be falsely addressed as an alteration to the number and physiological status of mitochondria in P-gp-positive cells [5]. Consistently, we observed real differences in JC-1 fluorescence between P-gp-positive (R and T) and P-gp-negative S cells by fluorescence flow cytometry (Figure 1A and Figure 2) and confocal microscopy (Figure 4). We tested VER, CSA and TQR, known P-gp inhibitors, for their abilities to eliminate differences in the JC-1 loading of P-gp-positive and P-gp-negative cells. Both of the most frequently used P-gp inhibitors VER and CSA failed to restore JC-1 double staining of P-gp-positive R and T cells to the extent observable in P-gp-negative S cells (Figure 1B). Similarly to VER or CSA in R and T cells, LP117, a novel commercially available drug-specific modulator of P-gp-mediated drug transport (http://www.medkoo.com/products/14425) was also not able to depress JC-1 transport in P-gp-positive neuroblastoma cells, even if this substance in the same cells induced the effective efflux blockage of vincristine, vinorelbine, actinomycin D, paclitaxel, and Calcein/AM [27]. In contrast to our results, CSA was able to restore JC-1 cell loading in several leukemia cell lines or cells obtained from leukemia patients [14]. This contradiction could be resolved by the following argumentation. A confusion between P-gp substrates and inhibitors exists [28], and many known P-gp inhibitors including VER and CSA are transportable substances for P-gp mediated transmembrane efflux, i.e., they could also be considered as substrates [29]. Therefore, these inhibitors induce transport depression of other P-gp substrates with at least partial competition with P-gp transport. Partial competition could be proposed from a detailed kinetic study of VER-induced activation of P-gp ATPase, which revealed the competitive inhibition of the ATPase by CSA and allosteric inhibition with an increasing Hill number for VER by vinblastine (another vinca alkaloid with strong structural similarity to VCR) [30]. JC-1 was described as a very sensitive probe for the estimation of P-gp efflux activity in the samples of leukemia patients [31], which indicated its high affinity as a P-gp substrate. This is consistent with the fact that both VER and CSA inhibited P-gp-mediated efflux of JC-1 with two-fold higher median inhibitory concentrations, as they inhibited Calcein/AM efflux in the NIH-3T3-G185 cell line presenting the gene product of human *ABCB1* [32]. Moreover, the effectiveness of P-gp inhibitors in the depression of JC-1 transport must also be related to the degree of P-gp expression in the tested cells. Consistent with this, recent results suggest that the transporter expression level crucially affects the intracellular levels of its substrates and thereby resistance to these substrates [33,34]. Thus, the affinity of the transportable substrate to P-gp, affinity of the inhibitor in the blockage of P-gp transport and amount of active P-gp molecules in the cell plasma membranes of the tested cells are involved in the final effectiveness of the P-gp efflux blockade for the respective inhibitor and substrate pair. Our P-gp-positive R and T cells express a large amount of P-gp (Supplementary materials, Figure S1), which is responsible for the several hundred times lower cell sensitivity to doxorubicin and VCR as P-gp prototypical cytotoxic drugs in comparison with P-gp-negative S cells [35]. In contrast to JC-1, the retention of either Calcein/AM or fluo-3/AM within R cells to an extent similar to that in S cells could be fulfilled by either CSA or VER. [23]. Taking all of the above facts together, it could be anticipated that CSA and VER, as inhibitors of P-gp efflux acting in a competitive manner [29], were not sufficiently potent inhibitors for the depression of a high affinity P-gp substrate efflux such JC-1 in R and T cells with massive P-gp expression. This idea is supported by the recently published assumption that the inhibition efficiency of competitive P-gp inhibitors depends on the P-gp expression levels, and cells expressing a high amount of this transporter require higher concentrations of inhibitor to achieve certain inhibitory effects [36].

To prove the latter idea, we have to show that a high affinity noncompetitive inhibitor of P-gp efflux activity is able to restore the R and T cells’ loading of JC-1. TQR, known as a potent, specific and noncompetitive P-gp inhibitor [37] that could be considered to be a high affinity inhibitor [38,39] and cannot be transported by P-gp [39], seems to fulfil these criteria. The data in Figure 1 and Figure 2 clearly show that TQR (in concentrations of 0.5 μM and higher) can restore JC-1 fluorescence in R and T cells to a similar extent as is observed for S cells. 

The ability of TQR to reverse P-gp-mediated vincristine resistance in R and T cells is documented in Table 1. The effect of TQR on the restoration of VCR resistance in R and T cells was concentration-dependent, similar to as was described for the effect of this P-gp inhibitor on the cytotoxicity of paclitaxel in human and mouse P-gp cell lines [39]. The TQR potency to reverse P-gp-mediated resistance to several P-gp-relative drugs including VCR was proven in a study when either in vitro or in vivo experiments were applied [40]. Moreover, in our experiments, the concentration of TQR that is sufficient for the effective reversal of the P-gp-mediated vincristine resistance of R and T cells is 0.5 μM (Table 1), i.e., the lowest fully effective concentration detected for the restoration of R and T loading by JC-1. We also proved that TQR is an inhibitor of P-gp-mediated Calcein/AM efflux in R and T cells (Figure 3). Non-fluorescent Calcein/AM, as an uncharged molecule, enters the intracellular space of cells by passive diffusion. Since this substance is a P-gp substrate, it could enter the intracellular space of P-gp-positive cells only when this transporter is effectively inhibited [19,23]. When it enters the intracellular space of cells, Calcein/AM is effectively de-esterified to green fluorescent free Calcein by cytosolic and lysosomal esterases [41]. Free Calcein is not a P-gp substrate, and as a charged substance, it could not diffuse across the plasma membrane, thus remaining in the intracellular space of cells [42,43]. Therefore, the measurement of the lack of Calcein retention within P-gp-positive cells and its restoration by P-gp inhibitors are often used an experimental strategy for the measurement of P-gp efflux activity [19] or the effectiveness of its inhibitors [44]. In our experiments, the retention of Calcein/AM within R and T cells is much less pronounced than in S cells, and TQR, in a concentration of 0.5–50.0 μM, restored Calcein retention within R and T cells to the extent observed in S cells. All of the above facts indicated that TQR blocked P-gp efflux activity effectively, resulting in the restoration of Calcein/AM as well as the JC-1 retention in R and T cells, thus reversing their vincristine resistance. However, TQR alone did not induce any significant cell death in S, R and T cells, as proven by the MTT test (Table 1) and the annexin-V/propidium iodide apoptosis/necrosis cytometric assay (Supplementary materials, Figure S3). TQR seems to interact in R and T cells exclusively with P-gp and depresses its efflux activity without any other effect on the processes responsible for the initiation and progression of cell death mechanisms. Therefore, this inhibitor represents a proper tool for the specific blockage of P-gp in P-gp-positive cells when JC-1 was used for the characterization of the membrane potential.

JC-1 was shown to be a substrate not only for P-gp but also for other two ABC transporters active in MDR: MRP1 and BCRP [45]. However, the expression of P-gp was detected as the prevalent molecular causality of the multidrug resistance of R cells [46]. Therefore, the contributions of MRP1 and BCRP to the net efflux of JC-1 are negligible in comparison with the massively expressed P-gp. An analogous situation could also be expected in T cells that expressed P-gp due to transfection with the human gene without any induction of the drug transporter due to the selective pressure of anticancer substances. In addition to the uncompetitive inhibition of P-gp drug efflux activity, TQR was described as a substrate and an inhibitor for breast cancer resistance protein [47]. The fact that S cell staining by JC-1 was not affected by TQR indicated that these parental cells did not express an effective amount of P-gp (which we documented in Figure S1 in the Supplementary materials) or BCRP, which we also proved in a set of unpublished experiments. In contrast to P-gp and BCRP, MRP1 is not effectively inhibited by TQR, even if this substance was used at a massive concentration of 50 μM [40]. However, the expression of MRP1 was detected to be much less pronounced than that of P-gp in R and T cells and was similar to that in S cells [48]. Taken together, we can conclude that in R and T cells, P-gp represents the correct barrier against the intracellular retention of JC-1, which is effectively reduced by TQR inhibitory action. The application of TQR for the achievement of JC-1 intracellular retention could also be effective in cells with the elevated expression of BCRP but not in cells with the elevated expression of MRP1. 

**Figure 5:**
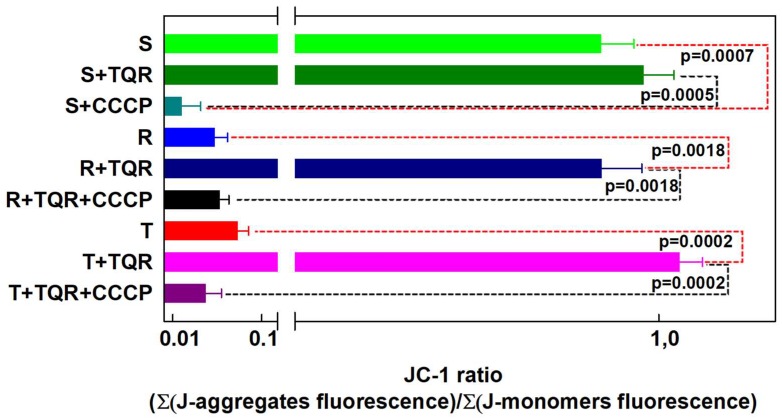
Mitochondrial membrane potential of S, R and T cells detected by the JC-1 ratio (J-aggregate fluorescence/J-monomer fluorescence). The sums of J-aggregate and J-monomer fluorescence from the measurements documented in Figure 2 were obtained from flow cytometry data and were used for accounting for the respective JC-1 ratios. The results represent the mean ± SD from three independent measurements. Statistical significance: Data connected by either red or black dashed lines differ significantly on mentioned probability levels p.

Confocal microscopy of JC-1-labeled S, R and T cells (the latter two in the presence TQR) revealed mitochondria double labeled (positive for both J-aggregates and J-monomers) to similar extents. Therefore, we may conclude that S, R and T cells contain uniform mitochondria with similar membrane potentials (ΔΨ). Το prove this idea, we used the JC-1 ratio between J-aggregate/J-monomer fluorescence, which was described as the measurement of mitochondrial ΔΨ [49]. The sums of the red and green fluorescences of S, R and T cells loaded with JC-1 in the absence or presence of 0.5 μM TQR and/or 50 μM CCCP obtained from cytometric measurements documented in Figure 2 were used for the calculation of the JC-1 ratio. Comparison of JC-1 ratios for S, R and T cells is documented in Figure 5. Almost identical JC-1 ratios were obtained for S, R and T cells in the presence of TQR. Thus, the membrane potentials of the mitochondria in S, R and T cells achieve similar values. In the absence of TQR, the JC-1 ratios for R and T cells were significantly lower than the JC-1 ratio in S cells. However, this difference is caused by alteration in the loading of R and T cells with JC-1 due to the P-gp efflux activity and does not reflect the status of mitochondria and their ΔΨ. The membrane potentials of S cells loaded with JC-1 as well as R and T cells loaded with JC-1 in the presence of 0.5 μM TQR could be efficiently reduced by the mitochondrial uncoupler CCCP at a concentration 50 μM according to Accuri application note [17].

In conclusion, it should be stressed that trouble in the characterization of mitochondria by a mitochondrial indicator such as JC-1 in P-gp-positive MDR neoplastic cells, which was advised by Cottet-Rousselle et al [5], could be eliminated by using a proper specific inhibitor such as TQR. The application of TQR or other substances belonging to the group of third generation of P-gp inhibitors such as elacridar, zosuquidar, laniquidar or OC144-093 could be usable tools for avoiding of P-gp efflux activity interference on measurements of the mitochondrial membrane potential using an appropriate fluorescent indicator.

## 4. Materials and Methods

### 4.1. Cell Cultivation Conditions and the Determination of P-gp Expression

The following L1210 cell variants were used in this study: (i) S, drug-sensitive parental cells obtained from the Leibniz-Institut DSMZ-Deutsche Sammlung von Mikroorganismen und Zellkulturen GmbH (Braunschweig, Germany) ACC-123; (ii) R, P-gp-positive drug-resistant cells overexpressing P-gp due to vincristine selection (Gedeon Richter Co., Budapest, Hungary) [15]; and (iii) T, P-gp-positive drug-resistant cells overexpressing P-gp due to stable transfection with the P-gp gene [16] from the Addgene plasmid 10957 (pHaMDRwt), a retrovirus encoding full-length P-gp cDNA [50]. The cells (S, R and T; 1 × 10^6^ cells) were cultured in 4 mL of RPMI 1640 medium with l-glutamine (1 mg/mL), 8% fetal bovine serum and 1 μg/mL gentamycin (all purchased from Gibco, Carlsbad, CA, USA) at 37 °C in a humidified atmosphere with 5% CO_2_. R cells were cultured for two passages without vincristine prior to each experiment. 

We periodically control P-gp expression at the mRNA and protein levels by RT-PCR, western blotting and confocal immunocytochemistry [51].

### 4.2. Flow Cytometry Detection of JC-1 Fluorescence in S, R and T Cells

After culturing, cells (10^5^) were harvested by centrifugation (5 min at 500× *g*) and then resuspended in 200 μL of RPMI medium without fetal bovine serum. To this mixture, the following P-gp inhibitors were added: (i) TQR (SelleckChem, Houston, TX, USA) to final concentrations of 0.05, 0.5, 5 and 50 μM in 1 μL of dimethyl sulfoxide (Sigma Aldrich, St. Louis, MO, USA); (ii) VER (Sigma Aldrich, USA) in final concentrations of 1 and 10 μM; and (iii) CSA (Sigma Aldrich) in final concentrations of 1 and 10 μM. Cells were left for 45 min in a CO_2_-incubator at 37 °C in a humidified atmosphere. S, R and T cells prepared similarly but without inhibitors were used as controls. After this incubation, JC-1 (5,5′,6,6′-tetrachloro-1,1′,3,3′-tetraethyl-imidacarbocyanine iodide, Sigma Aldrich) was added to a final concentration of 2.5 μM, and cells were shaken in the dark at 37 °C for 15 min. Afterwards, cells were counted in a BD Accuri C6 flow cytometer (BD Bioscience, San Jose, CA, USA) according to the protocol given in Accuri cytometers application note [17]. 

### 4.3. Confocal Microscopy of JC-1 Stained S, R and T Cells

Similarly processed cells as described in chapter 2.2 were additionally stained by adding DAPI (Sigma Aldrich, USA) to a final concentration of 10 mg/L to visualize the nuclei, and they were also used for confocal microscopy on a Nikon Eclipse Ti-E (Nikon, Tokyo, Japan). Samples were evaluated using 488 nm excitation and the registration of both green and red fluorescence. Samples were evaluated by using NIS-Elements software (Nikon CEE GmbH, C.R.).

### 4.4. Examination of the TQR Inhibitory Effect on P-gp Efflux Activity in S, R and T Cells using the Calcein/AM Assay

Initially, cells were incubated with TQR similar to as described in Section 2.2. Then, the P-gp transport activity was measured using a previously described protocol of a Calcein retention assay [23,43,52]. Cells were centrifuged (500× *g*), washed three times with PBS containing 0.2% BSA, and then resuspended in 500 μL of the same buffer. Calcein/AM (final concentration of 0.1 μM, Sigma-Aldrich, USA) was added directly to the buffer, and the samples were incubated for 20 min at 37 °C in a CO_2_ incubator. Propidium iodide (final concentration of 0.9 μM, Sigma-Aldrich, USA) was then incubated with the cells for an additional 10 min, and then, the cells were washed twice with ice-cold PBS. The fluorescence was measured using a BD Accuri C6 flow cytometer. Only viable propidium iodide-negative cells (greater than 92% in each case) were counted.

### 4.5. Measurement of the P-gp-Mediated Vincristine Resistance Reversal Induced by TQR

The cells (5 × 10^4^ cells/well) were cultured in the presence or absence of TQR (in a concentration range of 0.05–50.00 μM) with or without VCR (0.25 μM) in 96-well cell culture plates. TQR and VCR were directly added to 200 μL of culture media. After 48 h, the cell viability was assessed using the MTT assay, which was performed by adding MTT ([3-(4,5-dimethyldiazol-2-yl)-2,5 diphenyltetrazolium bromide]) to a final concentration of 0.25 mg·cm^−3^ per well. The cells were then incubated with MTT for 2 h. Next, the plates were centrifuged for 15 min (6000× *g*), and the cell sediment was extracted with dimethyl sulfoxide. The absorbance at 540 nm was measured using a multidetection plate reader Synergy H1 (BioTek Instruments, Winooski, VT, USA). 

### 4.6. Statistical Analysis and Data Processing

Numerical data are expressed as the mean ± SD of three independent measurements. Statistical significance was assessed using an unpaired Student’s *t*-test using SigmaPlot 8.0 software (Systat Software, Inc., San Jose, CA, USA).

## Figures and Tables

**Table 1:** Reversal of the P-gp-mediated VCR resistance of R and T cells by TQR.

		Control		TQR (0.05 μM)		TQR (0.50 μM)	
		VCR (0.25 μM)		VCR (0.25 μM)		VCR (0.25 μM)	
S cells	means ± SD	100.0 ± 14.7	10.5 ± 3.4	95.2 ± 12.8	11.5 ± 6.5	95.2 ± 10.3	11.8 ± 5.1
Student‘s *t*-test	*t*-value	11.9		11.7		13.2	
	*p*-value	2.1 × 10^−5^		2.4 × 10^−5^		1.2 × 10^−5^	
R cells	means ± SD	100.0 ± 17.9	95.6 ± 9.2	103.4 ± 9.3	67.8 ± 9.5	95.8 ± 18.0	11.3 ± 5.6
Student‘s *t*-test	*t*-value	0.43		5.4		9.0	
	*p*-value	0.68		1.7 × 10^−3^		1.1 × 10^−4^	
T-cells	means±SD	100.0 ± 17.2	97.2 ± 15.1	104.4 ± 16.6	36.5 ± 12.0	104.2 ± 15.2	11.5 ± 4.3
Student‘s *t*-test	*t*-value	0.24		6.6		11.7	
	*p*-value	0.82		5.6 × 10^−4^		2.3 × 10^−5^	

The cell viability was measured by the MTT test. The MTT signals obtained for S, R and T cells in the absence of vincristine (VCR) and tariquidar (TQR) were arbitrarily taken as 100%. All data were measured in medium containing the same amount of dimethyl sulfoxide that is necessary for TQR application. The green highlighted part represents the sensitivity of S cells to VCR independent of the presence of TQR. The red highlighted part represents the resistance of R and T cells to VCR in the absence of TQR. The blue highlighted part represents partial reversal of the resistance of R and T cells to VCR by TQR at a concentration of 0.05 μM. The grey highlighted part represents complete reversal of the resistance of R and T cells to VCR by TQR at a concentration 0.50 μM.

## Supplementary Materials

Supplementary materials can be found at http://www.mdpi.com/1422-0067/19/7/1985/s1.

## Abbreviations

BCRP	Breast cancer resistance protein, ABCG2 protein
CCCP	Carbonyl cyanide 3-chlorophenylhydrazone
CSA	Cyclosporine A
DAPI	4′,6-Diamidino-2-phenylindole
ΔΨ	Mitochondrial membrane potential
JC-1	5,6-Dichloro-2-[(E)-3-(5,6-dichloro-1,3-diethylbenzimidazol-3-ium-2-yl)prop-2-enylidene]-1,3-diethylbenzimidazole iodide
MDR	Multidrug resistance
MRP(1-3)	Multidrug resistance-associated proteins (1-3), ABCC (1-3) proteins
MTT	([3-(4,5-Dimethyldiazol-2-yl)-2,5 diphenyltetrazolium bromide])
P-gp	P-glycoprotein, ABCB1 protein
R	P-gp-positive variant of L1210 cells obtained by selection with vincristine
S	P-gp-negative L1210
T	P-gp-positive variants of L1210 cells obtained by transfection gene encoding P-gp
TQR	Tariquidar
VCR	Vincristine
VER	Verapamil
