# Supplementary material for: Detection of the Mitochondrial Membrane Potential by the Cationic Dye JC-1 in L1210 Cells with Massive Overexpression of the Plasma Membrane ABCB1 Drug Transporter
Source: Int J Mol Sci. 2018 Jul 7;19(7):1985. doi: 10.3390/ijms19071985 (PMC6073605; doi:10.3390/ijms19071985)
Supplement: Supplementary file 1 [file ijms-19-01985-s001.pdf]

Supplementary files

Elefantova et al., "Detection of the Mitochondrial Membrane Potential by the Cationic Dye JC-1 in L1210 Cells with Massive Overexpression of Plasma Membrane ABCB1 Drug Transporter."

1. Methods of P-glycoprotein expression measurements in S, R and T cells.

1.1. RT-PCR and qRT-PCR

Total RNA was isolated from the S, R and T cells using TRIzol reagent (Life Technology, Slovakia) according to the manufacturer's instructions. A reverse transcription (RT) was performed with 2 μg of total DNAse I (Thermo Scientific, Germany)-treated RNA and the RevertAidTM H Minus First Strand cDNA Synthesis Kit (Thermo Scientific, Germany) according to the manufacturer's protocol. PCR was performed in a total volume of 25 μl, consisting of 4 μl of RT mixture, 1x PCR buffer, 1.5 mmol/L $MgCl_2$, 0.2 mmol/L dNTP, 1 pmol of each specific gene primer set and 0.3 U of DyNAzyme II DNA polymerase (Finnzymes OY, Finland) in the buffer provided by the manufacturer. After treating the samples at 94°C for 3 min to inactivate the reverse transcriptase, they were subjected to 35 cycles of denaturation (95°C, 45 s.), annealing (57°C, 30 s.- P-gp from *Mus musculus* and *Homo sapiens*; 58°C, 30 s.- GAPDH, and extension (72°C, 90 s.) and then a final extension at 72°C for 10 min. The PCR products were separated on a 1.7% agarose gel (Invitrogen, Life Technology, Slovakia), and specific bands were visualized using ethidium bromide with a Typhoon 9210 imaging system (GE Healthcare, USA, formerly Amersham Biosciences) and normalized to the band intensity of GAPDH.

Semiquantitative real-time PCR was performed in a total volume of 20 μl using Maxima SYBR Green/ROX qPCR Master Mix (2x) (Thermo Scientific, Germany) and 250 nmol/L of each primer. Amplification and detection were performed with an ABI Prisma 7900HT detection system (Applied Biosystems, USA) under the following conditions: 95°C for 10 min, 40 cycles of denaturation (95°C, 15 s.), annealing (57°C, 30 s. – P-gp from *Mus musculus* and *Homo sapiens*; 58°C, 30 s. – GAPDH), and extension (72°C, 30 s.) and then a final melting curve analysis. The sequences of the primers employed in this study are as follows: GAPDH, F: 5′-TGA ACG GGA AGC TCA CTG G-3′, R: 5′-TCC ACC ACC CTG TTG CTG TA-3′; P-gp from Mus musculus, F: 5′-GGC TGT TAA AGG TAA CTC C-3′, R: 5′-TGT TCT CTT ATG AAT CAC GTA-3′; P-gp from Homo sapiens, F:5′-AGA CAT GAC CAG GTA TGC-3′, R: 5′- CTC CTG TCG CAT TAT AGC-3′. The data are expressed as the relative level of each mRNA normalized to that of the housekeeping gene GAPDH.

1.2. Compensation of JC-1 cytometric data.

According to Accuri cytometers Application note (see ref. [17]) compensation of cytometric data is necessary for correct data evaluation. Software compensation of data was processed (using BD Accuri C6 software) in range 10-25%. Minimal compensation that assured good resolution was used for evaluation. Starting with compensation at value between 11% and 12% represents recommended operation on the C6 Flow Cytometers in this note (see ref. [17]). Diminution of mitochondrial membrane potential was induced by mitochondrial uncoupler carbonyl cyanide 3-chlorophenylhydrazone (CCCP) at recommended concentration 50 μM concentration in Accuri cytometers Application note [17].

1.3. Western blotting.

After incubation, the cells were harvested, and crude membrane fractions were prepared with a ProteomeExtract Subcellular Proteome Extraction Kit (Calbiochem, USA) according to the manufacturer's instructions. The proteins from the samples were separated by sodium dodecyl sulfate-polyacrylamide electrophoresis using 8% polyacrylamide gels. The proteins were then transferred by

electroblotting to a PVDF membrane. P-gp was detected using the c219 anti-P-gp monoclonal antibody (Calbiochem, USA). A secondary anti-mouse antibody conjugated to horseradish peroxidase (GE Healthcare, USA) was used for detection. To provide an internal control for protein loading, a rabbit polyclonal antibody against GAPDH (Santa Cruz Biotechnology, USA) was used as a primary antibody, and goat anti-rabbit IgG conjugated to horseradish peroxidase (Santa Cruz Biotechnology, USA) served as a secondary antibody. The protein bands were visualized using an ECL detection system (GE Healthcare, USA) and a CF 440 imaging system (Kodak, USA).

1.4. *Immunofluorescence confocal microscopy.*

After cultivation, the cells were washed, resuspended in PBS, and transferred onto poly-L-lysine cover glasses (Menzel Glaser, Germany). The bound cells were washed twice with PBS and then fixed and permeabilized with methanol at -20°C for 20 min. The cells were again washed with PBS and then blocked by incubation with 1% BSA in PBS for 1 h at 37°C. Next, they were incubated with the c219 anti-P-gp antibody for 1 h at 37°C in PBS containing 1% BSA. After the primary antibody incubation, the cells were washed twice with PBS containing 1% BSA. Then, the cells were incubated with FITC-conjugated goat anti-mouse antibody (Calbiochem, USA) in PBS containing 1% BSA for 1 h at 37°C and then washed twice in PBS containing 1% BSA. Finally, the coverslips were mounted onto slides with a mounting medium (80% glycerol) and analyzed using a confocal laser scanning microscope (LSM 510 META; Carl Zeiss, Germany).

1.5. *Measurement of cell viability using annexin-V/propidium iodide assay.*

Cells were incubated with TQR similar to the description in chapter 2.2. The proportions of apoptotic and necrotic cells were then detected using a fluorescein isothiocyanate-labeled annexin-V (FAV) and propidium iodide (PI) kit (Calbiochem, USA). According to the procedure described by the manufacturer, the cells were washed twice with PBS and gently resuspended in binding buffer (obtained from the manufacturer) containing 0.5 μg/ml FAV. The mixture was then incubated in the dark for 15 min at room temperature and centrifuged (500×g for 15 min). The resulting sediments were resuspended in binding buffer, propidium iodide (final concentration 0.6 μg/ml) was added to each sample, and the samples were analyzed by flow cytometry using an BD Accuri C6 flow cytometer.

2. Supplementary results

2.1. Expression of P-gp in R and T cells

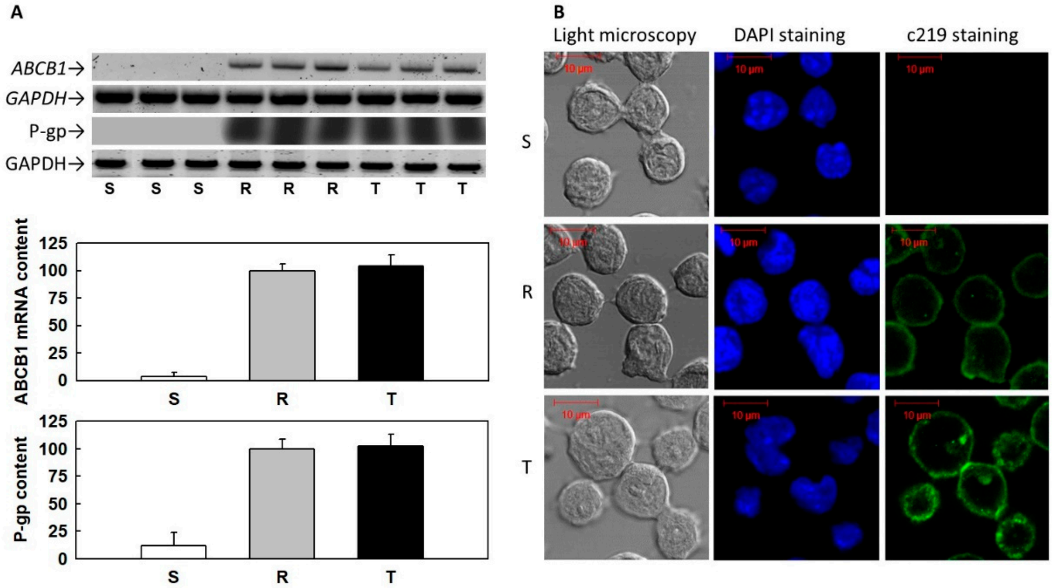

Figure S1. Expression of P-glycoprotein in R and T cells. Panel A: Cell contents of *ABCB1* gene transcript encoding P-gp and P-gp protein estimated by RT-PCR and Western blotting. Upper part: Documentation of respective gel with PCR product and blot membrane. The results were measured in triplicate independent measurements and are expressed in % relative to GAPDH as housekeeper. Lower part: Quantification of mRNA content by qRT-PCR and Western blot densitometry. Data represent the mean±S.E.M. from three independent measurements. Panel B: Immunofluorescence staining of P-gp in cells with anti-P-gp antibody c219. Nuclei were stained by DAPI. Data are representative of three independent experiments. (Experimental data were obtained during the diploma thesis project of Jan Stetka at the Institute of Molecular Physiology and Genetics, Centre of Bioscience SAS)

Conclusion: Both R and T cells express massive amounts of *ABCB1* gene transcript and P-gp protein that is localized predominantly on the cell surface. In contrast, parental cells did not exert such a pronounced P-gp expression.

2.2. *Compensation of JC-1 cytometric Data*

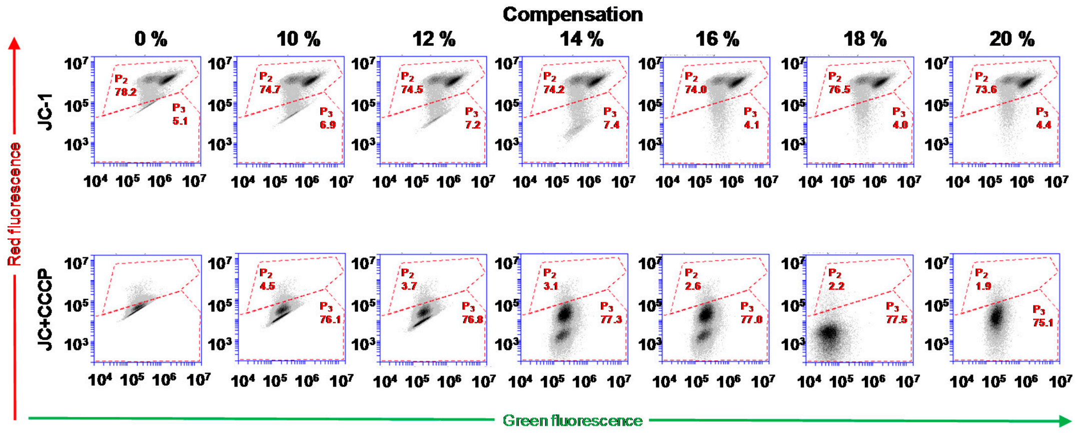

Figure S2. Compensation of JC-1 cytometric data. Data for S cells are documented on figure without compensation and with compensation in range 10-20%. Mitochondrial membrane potential was diminished by addition of Carbonyl cyanide 3-chlorophenylhydrazone (CCCP) to final concentration 50 μM. Compensation 18% gave similar results than 20% and 25% (latter not shown) therefore this compensation was used in data evaluation. Similar measurements were made also for R and T cells in the absence and presence of tariquidar and compensation 18% was always optimal.

Conclusion: Compensation 18% gave optimal results, which were not changed by further increasement to 20%. Similar procedure was applied also for R and T cells and compensation 18% always gave optimal results.

2.3. Effect of tariquidar on S, R and T cell viability

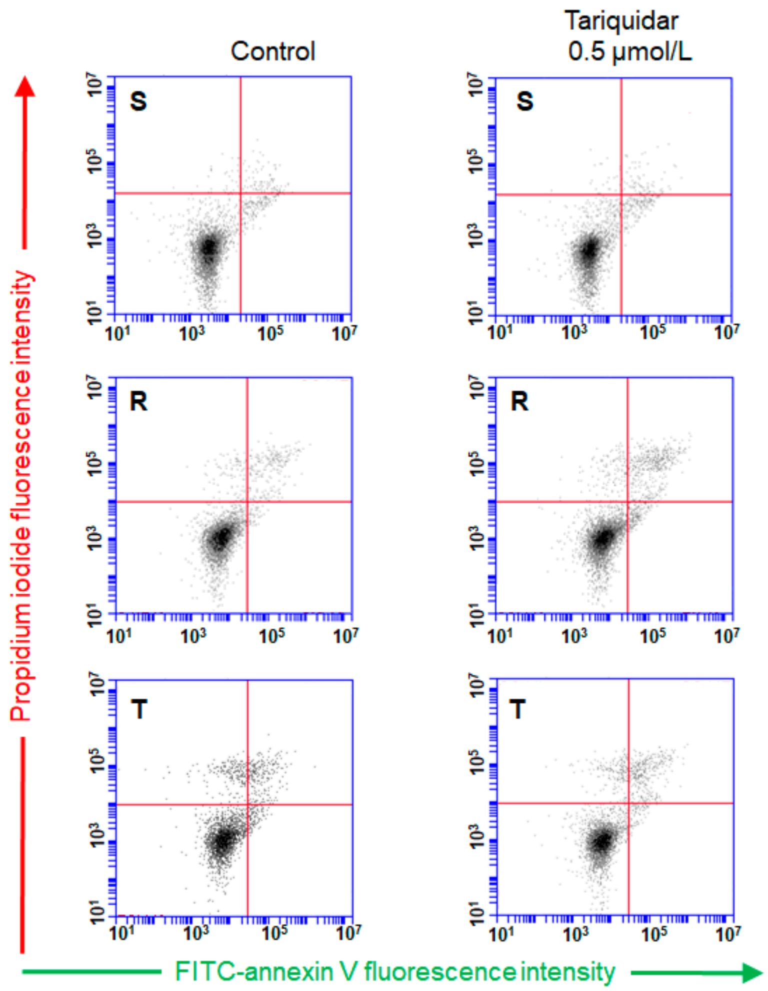

Figure S3. Detection of apoptosis and necrosis in S, R and T cells after incubation of cells with tariquidar by the FAV and PI apoptosis-necrosis kit. Lower-left quadrant: viable cells unlabeled by FAV and PI; lower-right quadrant: early-apoptotic cells labeled by FAV only; upper-left quadrant: early-necrotic cells labeled by PI only; upper-right quadrant: late-apoptotic/necrotic cells labeled by both FAV and PI. Data are representative of three independent measurements.

Conclusion: Incubation of S, R and T cells with TQR alone (at concentration 0.5 µM) did not induce cells entering into an apoptotic or a necrotic mode of cell death.
